# Sewage Sludge as a Tool in Limiting the Content of Trace Elements in *Avena sativa* L. on the Soil Polluted with Diesel Oil

**DOI:** 10.3390/ma14144003

**Published:** 2021-07-17

**Authors:** Mirosław Wyszkowski, Jadwiga Wyszkowska, Agata Borowik, Natalia Kordala

**Affiliations:** 1 Department of Agricultural and Environmental Chemistry, University of Warmia and Mazury in Olsztyn, Łódzki 4 Sq., 10-727 Olsztyn, Poland; natalia.kordala@uwm.edu.pl; 2 Department of Soil Science and Microbiology, University of Warmia and Mazury in Olsztyn, Łódzki 3 Sq., 10-727 Olsztyn, Poland; jadwiga.wyszkowska@uwm.edu.pl (J.W.); agata.borowik@uwm.edu.pl (A.B.)

**Keywords:** diesel oil, stabilised sewage sludge, trace elements in oat

## Abstract

The aim of the research was to determine the effect of soil contamination with diesel oil (0; 5; 10 and 15 cm^3^ kg^−1^ of soil) on the content of trace elements in the aboveground parts of oat (*Avena sativa* L.). Stabilised sewage sludge was used to mitigate the likely negative impact of diesel oil on the plant. Growing soil contamination with diesel oil had a significant impact on the content of trace elements in the aboveground biomass of oat. In the series without sewage sludge, the contents of the analysed elements, except for chromium, zinc, copper and cobalt, were positively correlated with the increasing doses of diesel oil. The largest increase in the content was recorded in the case of manganese. The sewage sludge used to reduce the influence of diesel oil on the chemical composition of oat had a positive effect on the content of the analysed trace elements. Compared to the series without the addition of a stabilised sewage sludge, it contributed to a reduction in the average content of chromium, nickel, copper, manganese and cobalt in the aboveground parts of oat plants. No significant effect of the applied remediation treatment was noted for cadmium, and the results were equivocal for iron.

## 1. Introduction

The rapid growth of industry and the widespread extraction and consumption of petroleum oil are among the chief causes of environmental contamination with hydrocarbons originating from petroleum products [1]. The accumulation of petrochemicals in the environment poses a direct threat to human health and to ecosystems [2]. These products contain a mixture of organic substances able to produce mutagenic and cancerogenic effects [3]. Soil pollution with crude oil and its derivatives induce significant changes in the soil’s biological parameters, such as soil enzymatic activity or number of soil microorganisms [4,5]. As well as deteriorating soil fertility [6], contamination with petroleum has a negative influence on the growth and development of crops [7,8,9]. Under such conditions, the natural cycling of elements in nature and their uptake by plants are distorted, which leads to changes in the content of macronutrients and trace elements in plants [10].

Trace elements are natural components of the environment, where their concentrations depend on the origin and chemical composition of the parent rock as well as the intensity of geological and soil-formation processes [11,12]. However, many elements enter soil in elevated quantities due to human activity, which turns the soil into a reservoir of these elements and, in certain circumstances, an uncontrolled source of such elements to living organisms [13]. We can distinguish among trace elements micronutrients essential for plant organisms, such as copper (Cu), zinc (Zn) and manganese (Mn), as well as useless elements, whose physiological role has not been completely elucidated yet, for example lead (Pb) and cadmium (Cd) [14,15]. What all trace elements share is that in high concentrations they are toxic to biotic elements of the environment [16]. Soil contamination with trace metals is most often caused by copper, nickel (Ni), cadmium, zinc, chromium (Cr) and lead [17].

Soil polluted with petroleum substances is exposed to an increase in the content of trace elements and other contaminants up to their toxic levels to living organisms [18]. Even a few or a few dozen cm^3^ of oil per kg of soil (depending on the plant species) limits the growth and development of plants and generally increases the concentration of elements and contaminants in plants. Their uptake by plants from the substrate through the roots causes chlorosis, inhibition of the growth of plants, decreased yields and lower uptake of nutrients, disturbed metabolism of plants and reduced biological nitrogen fixation by plants in the family Fabaceae [19,20]. It has been observed that an elevated content of trace elements in soil evokes changes in the number, community composition and activity of soil microorganisms [21].

The elements absorbed by plants from the soil are mostly retained in the roots [12,22]. Constant emission of trace elements from various sources causes a continual increase in their content of soil and in air, in consequence being responsible for their excessive migration to aboveground parts of plants [23,24]. Some higher plant species can accumulate trace elements in tissues without manifesting evident disease symptoms, which raises the risk that toxic quantities of trace elements may be included in human and animal diets [25].

The restoration of degraded soils and industrial areas can include treatments with organic waste, for instance sewage sludge [26]. This is organic material with beneficial fertilising properties, which is a byproduct of biological wastewater treatment [27]. Sewage sludge is rich in organic matter and macronutrients essential for the growth of plants [28,29], which can also improve the microbiological and enzymatic activity of the soil [27,30]. The application of municipal sewage sludge in the cultivation of cereals enables the recycling of the nutrients contained in this waste, especially phosphorus. It is estimated that around 60% of the phosphorus which reaches wastewater treatment plants remains in the sewage sludge [31]. Sewage sludge can be also an alternative to conventional fertilisers [32]. Fertilisation with sewage sludge has a lasting effect on the microbiological activity in soil’s accumulation layer [33]; it helps to maintain the right amount of humus in soil and its raises the enzymatic activity of the soil as well as the synthesis of humic acids [34,35]. According to Wyszkowska et al. [36], the bioavailability of trace elements in soils poor in humic acids is much higher. As the disposal of sewage sludge is difficult and its storage not possible, its use in natural processes is ever more justified [37].

The aim of this study was to determine the influence on soil contamination with diesel oil on the content of trace elements in aboveground parts of *Avena sativa* L. Another purpose was to determine the usefulness of stabilised sewage sludge as a material limiting the negative effects of the abovementioned petroleum products on the chemical composition of the test plant.

## 2. Materials and Methods

### 2.1. Assumptions Underlying the Experiment

A pot experiment was carried out in a greenhouse at the University of Warmia and Mazury in Olsztyn (north-eastern Poland), on soil with the textural composition of sandy loam (sand >0.05 mm 78.08%, silt 0.02–0.05 mm 20.46%, and clay <0.002 mm 1.46%) according to the soil texture classification by the United States Department of Agriculture. The experiment was set up to examine the impact of soil pollution with diesel oil in doses of 0, 5, 10 and 15 cm^3^ kg^−1^ d.m. of soil on plants, and the effects of an application of stabilised sewage sludge in doses of 0, 4, 8 and 12 g kg^−1^ of soil to alleviate the influence of the pollutant. The stabilised sewage sludge and diesel oil were added once before start of experiment. The following macronutrients were added to each pot: nitrogen in an amount of 112 mg [CO(NH_2_)_2_], phosphorus—39 mg [KH_2_PO_4_], potassium—112 mg [KH_2_PO_4_ + KCl] and magnesium—15 mg kg^−1^ d.m. of soil [MgSO_4_·7H_2_O]. A batch of soil weighing 2.8 kg was mixed with the diesel oil, sewage sludge and mineral fertilisers, after which it was placed in polyethylene pots. Next, oat plants (*Avena sativa* L.) of the variety Furman were seeded. Twelve plants were grown per pot. The experiment was carried out in four replications. Throughout the experiment, the soil moisture was maintained on a constant level (60% of water capillary capacity). The soil moisture was monitored every day. Oat harvest was carried out at the fully emerged inflorescence stage (BBCH 59). During harvest, soil samples for analysis were collected.

Diesel oil was bought at an Orlen petrol station. Diesel oil (d = 845 g dm^−3^) contained aromatic polycyclic compounds, mainly three-ringed ones. Detailed characteristics of Verva—type B diesel oil (DO) can be found on the website www.orlen.pl.

### 2.2. Characteristics of Soil and Sewage Sludge

The soil material was collected from the organic topsoil horizon of a typical brown earth (Eutric Cambisol), from a field at the Experimental Station in Tomaszkowo (NE Poland, 53.7161° N, 20.4167° E). The stabilised sewage sludge obtained from the Wastewater Treatment Plant in Tyrowo was determined not to contain specific DNA of *Salmonella* sp., or live eggs of intestinal parasites *Ascaris* sp., *Trichuris* sp., *Toxocara* sp. The physiochemical properties of both the soil and sewage sludge used in this experiment are presented in Table 1.

### 2.3. Research Procedures Applied

Samples of plant material collected during the harvest of oat were cut, dried, ground and stored in plastic containers. The prepared plant material was wet-digested (in three replications) in analytical grade concentrated nitrogen acid (density of 1.40 g cm^−3^) in Teflon Xpress vessels placed in a microwave oven MARS 6—CEM Corporation, Matthews, NC, USA (CEM, 2016), based on US-EPA3051A method [38]. The digested samples were submitted to determinations of the content of cadmium, lead, chromium, nickel, zinc, copper, manganese, iron and cobalt, using flame atomic absorption spectrometry (FAAS) in an air–acetylene flame on an absorption spectrometer SpectrAA 240FS - VARIAN, Austria [39]. The Fluka company standard solutions (Cd 51994, Pb 16595, Cr 02733, Ni 42242, Zn 188227, Cu 38996, Mn 63534, Fe 16596, Co 119785.0100) and the Certified Analytical Reference Material (NCS ZC 73030) in laboratory analyses were used.

Basic characteristics of the soil prior to the experiment were determined as follows: particle size distribution with the aerometric method [40], pH in 1 M KCl with the potentiometric method [41], hydrolytic acidity and cation exchange capacity with the Kappen method [42], and the content of total nitrogen with the Kjeldahl method [43], organic carbon with the Tiurin method [44], available forms of phosphorus and potassium with the Egner–Riehm method [45], and magnesium with the Schachtschabel method [46], dry mass with drying method [39].

### 2.4. Statistical Analysis

The normality of data distribution was verified with the Kruskal–Wallis and Shapiro–Wilk tests. The results were compared in ANOVA and Honestly Significant Difference (HSD) Tukey test. Homogeneous groups were identified at a significance level of *p* ≤ 0.01. Percentage contribution of dependent and independent variables was calculated based on the η2 coefficient using the analysis of variance (ANOVA) at a significance level of *p* ≤ 0.01. Additionally, Pearson correlation coefficients were computed for the analysed parameters. Results concerning heavy metal content in the aboveground parts of oat were subjected to the principal component analysis (PCA) using multidimensional explorative techniques. All calculations were supported by the software package Statistica [47].

## 3. Results

### 3.1. Trace Elements Content in Plants

The content of trace elements in aboveground parts of the oat plants was affected by both the applied level of soil pollution with diesel oil and the application of sewage sludge to soil.

In the series without sewage sludge, the contamination of the soil with diesel oil resulted in a higher content of cadmium, lead, nickel, iron and manganese in the aboveground biomass of the test plant (Table 2, Table 3 and Table 4). The biggest changes were noted in the case of lead and manganese. The highest dose of diesel oil (15 cm^3^ kg^−1^ d.m. of soil) induced an increase in the content of lead by 74% (r = 0.954) while the content of manganese in aboveground parts of oat nearly trebled (r = 0.999) in comparison with the control treatment (not polluted with diesel oil). The content of cadmium, nickel and iron in the analogous objects changed less. High doses of diesel oil caused an increase in the content of cadmium by 23% (r = 0.943), nickel by 10% (r = 0.980) and iron by 18% (r = 0.748) in the aboveground parts of oat relative to the control.

Under the influence of soil contamination with diesel oil in the series without the application of sewage sludge, the content of copper, zinc, chromium and cobalt in the aboveground parts of oat was decreased (Table 2, Table 3 and Table 4). The biggest decrease (by nearly four-fold, r = −0.977) was noted for the copper content. The content of zinc, chromium and cobalt in the aboveground parts of oat decreased by 30% (r = −0.758), 29% (r = −0.687) and 18% (r = −0.955) at the most, respectively, in comparison with the control. 

The application of sewage sludge to soil had a slightly significant or considerable effect on the content of trace elements in the aboveground parts of oat (Table 2, Table 3 and Table 4). The sewage sludge caused a reduction in the content of most trace elements (except lead, zinc and, in some cases, nickel) in the aboveground biomass of the test plant grown on soil contaminated with diesel oil. A small effect was observed in the case of cadmium, while changes in the content of iron were not consistent. The biggest variation was observed in the content of manganese, which decreased on average by 3% to 12% in response to the application of the sewage sludge to the soil compared to the series without this neutralising material. Soil remediation with sewage sludge caused a statistically significant increase in the zinc and lead content in the aboveground biomass of oat. The highest increase in the content of zinc (by 15%) and lead (by 16%) in the aboveground parts of oat was recorded in the series with sewage sludge added in an amount of 8 g kg^−1^ d.m. of soil compared to the control object (without sewage sludge).

### 3.2. Statistical Analysis

**Figure 1:**
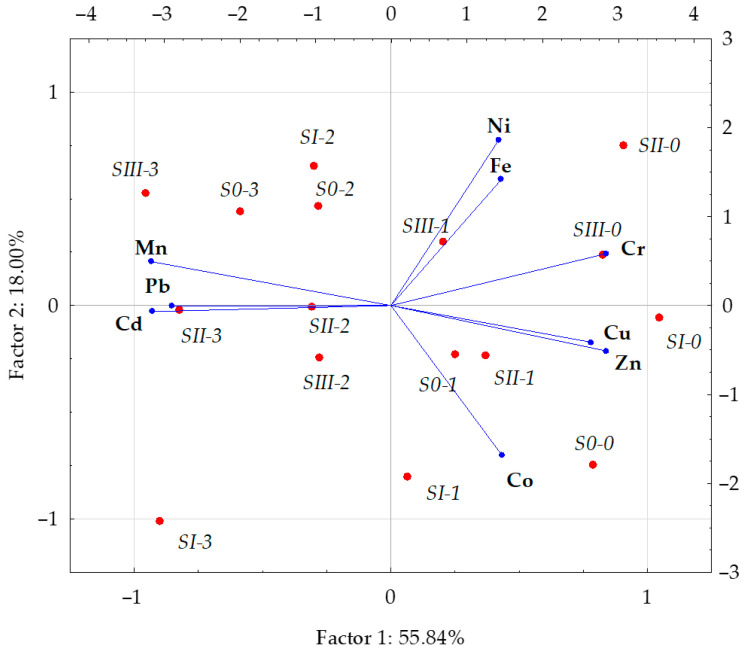
Content of trace elements in the aboveground parts of oat (*Avena sativa* L.) illustrated with the PCA method. Key: vectors represent analysed variable (content of Cd, Pb, Cr, Ni, Zn, Cu, Mn, Fe and Co); points show the samples with elements (*S0* - without sewage sludge, *SI* - 4 g, *SII* - 8 g, *SIII* - 12 g sewage sludge per kg of soil; 0–0 cm^3^, 1–5 cm^3^, 2–10 cm^3^, 3–15 cm^3^ diesel oil per kg of soil).

The correlation coefficients, shown in Table 5, and the results of the PCA (Figure 1) confirm the presence of strong correlations between individual trace elements in the aboveground biomass of oat. The strongest positive correlation was determined between lead versus cadmium and manganese as well as between copper and zinc. The strongest negative correlation appeared between copper and manganese, while a weaker positive correlation was noticed between zinc and chromium versus cadmium. The principal component analysis revealed changes in the content of trace elements in the aboveground biomass of oat effected by the applied doses of diesel oil and sewage sludge (Figure 1). The first cluster of trace elements, that is cadmium, lead, chromium, zinc, copper and manganese, provided 55.84% of the information on the chemical composition of oat comprised in the input variables, whereas the second group, consisting of nickel, iron and cobalt, explained 18.00% thereof. Having compared the lengths of the vectors of the analysed elements, it was concluded that the vectors of manganese, cadmium, nickel and zinc were longer than the other ones, which indicates their greater contribution to the correlation of the set of data. The scattering of points in Figure 1 suggests that the application of sewage sludge to soil contaminated with diesel oil has a strong effect on the some analysed elements in the aboveground parts of oat.

**Figure 2:**
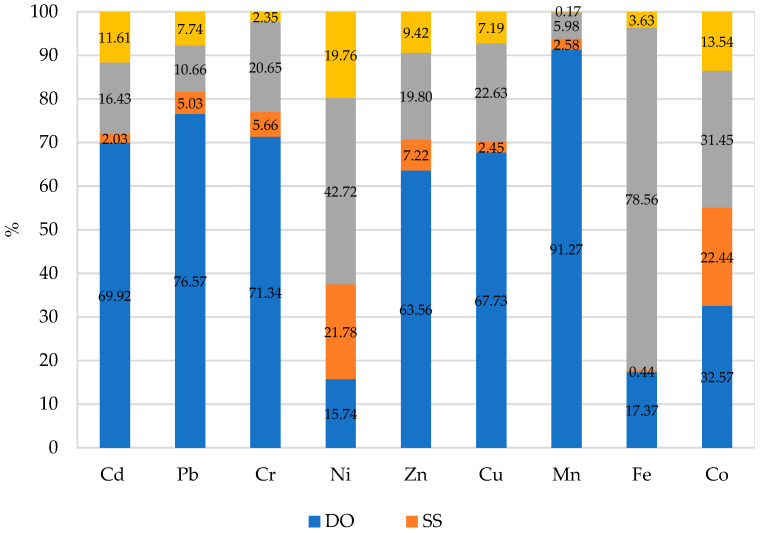
Percentage contribution of variable factors according to the content of trace elements in aboveground parts of oat (*Avena sativa* L.): DO—diesel oil dose, SS—sewage sludge dose.

The determination of the percentage of observed variation with the help of the η2 coefficient, using the ANOVA method, demonstrated that the content of trace elements in oat was most strongly dependent on a dose of diesel oil, especially in the case of chromium, lead and manganese (71.34%, 76.57% and 91.27% of the contribution of the respective variable) (Figure 2). A considerable contribution of diesel oil to the change in the content was also observed for zinc (63.56%), copper (67.73%) and cadmium (69.92%). Much lower contributions were recorded for the other elements, ranging from 15.74% for nickel to 32.57% for cobalt. In samples of aboveground biomass of oat collected from the objects with the application of sewage sludge, its contribution was much lower and ranged from 2.03% (cadmium) to 32.57% (cobalt). The application of sewage sludge had a much stronger effect on the content of copper (21.78%) and cobalt (22.44%).

## 4. Discussion

### 4.1. The Effect of Diesel oil Contamination on Plants

The development of industry, transportation and urbanisation has contributed to the pollution of the natural environment with petroleum substances [3]. The main sources of such substances are road accidents, leaks in underground storage tanks, the uncontrolled operation of landfills, petroleum-derived sludge, drill cuttings polluted with crude oil and corroded pipes [48].

Diesel oil is a complex mixture of hydrocarbons as well as low molecular alkanes and polycyclic aromatic hydrocarbons [49]. It also contains sulphur, nitrogen and oxygen as well as trace elements, such as iron, lead, copper, chromium, zinc and cadmium [50,51]. Contamination of the soil with diesel oil leads to the accumulation of nutrients (K, P, Ca, Mg, Na) and trace elements (Mn, Pb, Zn, Fe, Co, Cu) in soil and, subsequently, their translocation into plant tissues [52,53,54]. Although some trace elements in small quantities are essential micronutrients for plants, in high concentrations they can cause metabolic disorders and inhibit the growth of most plant species [1,55].

Soil pollution with petroleum derivatives has a negative effect on the growth of plants, number of roots and their length [56]; it decreases the number of leaves and the content of nitrogen and phosphorus in soil, and it reduces transpiration, photosynthesis and transport of nutrients [57]. Diesel oil components cause changes in the content of macro- and microelements in plants, typically raising the level of trace elements [58], which may have an indirect influence of their content in plants. Some researchers [6,10,56] have also demonstrated that products derived from crude oil cause an increase in the content of cadmium, lead, copper and manganese in soil. This can explain the elevated content of cadmium, lead and manganese in aboveground parts of oat plants detected in this study.

A decrease in the content of copper and higher accumulation of manganese in aboveground parts of wheat (*Triticum aestivum* L.) following the application of diesel oil in a dose of 18 g kg^−1^ d.m. of soil were confirmed by Rusin et al. [22]. In the same conditions, these authors noted a lack of a statistically significant influence of diesel oil on the content of lead, zinc and cadmium in the analysed parts of the plant.

In our study, a positive correlation between the soil pollution with diesel oil and the content of trace elements was demonstrated for cadmium, lead, nickel, iron and manganese. The results presented in this article are comparable with the ones reported by Shukry et al. [59], who evaluated the effect of petroleum on the chemical composition of jojoba plants (*Simmondsia chinensis*) and showed that the increasing concentration of petroleum in soil (1, 2 and 3% *v*/*w*) caused a higher accumulation of copper, manganese, cadmium and lead as well as a decreased content of zinc both in shoots and in roots of the test plants. A possible cause of the higher content of the mentioned elements in organs of plants grown on soil polluted with petroleum-derived compounds could be the acidifying effect of the latter. Akpan and Udoh [60] showed a decline in the soil pH from 4.90 to 4.35 two weeks after the application of diesel oil at the lowest level of pollution (5.06%). The cited authors noted the same tendency after 18 weeks. Similar changes in soil acidity were observed by John et al. [61]. The soil reaction is considered to be one of the principal factors influencing the quantities of phytoavailable forms of trace elements [62]. A decrease in the soil reaction to slightly acidic and acidic elements stimulates the mobility of available forms of trace elements, thereby raising the risk of increasing the indicator of their translocation to shoots [63,64].

A decrease in the content of chromium, zinc, copper and cobalt in aboveground parts of oat in response to the soil pollution with diesel oil was noted in this experiment at nearly all the applied doses of the pollutant. An exception was the dose of diesel oil equal 5 cm^3^ kg^−1^ d.m. of soil, which caused a statistically significant increase in the cobalt content. Similar results were obtained by Sivitskaya and Wyszkowski [65], who demonstrated that the increasing level of soil pollution with heating oil, which has similar characteristics to those of diesel oil, caused a decrease in the content of copper and zinc but an elevated accumulation of nickel in maize. The cited experiment, in the same way as the one reported herein, failed to reveal consistently directed changes in the content of iron in the aboveground biomass of the test plant due to the pressure of crude oil derivatives in soil. A similar effect of diesel oil on the content of zinc and copper accompanied by the accumulation of lead in leaves of broad bean was noted by Gospodarek and Nadgórska-Socha [66].

### 4.2. The Impact of Sewage Sludge Application on Trace Elements Content in Plants on Soil Contaminated with Diesel Oil

Organic material, either present in the soil environment or brought into the soil by natural or organic fertilisers, has an effect on the immobilisation and stabilisation of trace elements in soil, thus helping to limit quantities of their phytoavailable forms [67], as a result of which the yields harvested from such soil have a smaller content of trace elements [12]. By binding specific trace elements into complex compounds, organic matter changes their solubility and lowers desorption into the soil solution as well as mobility in soil [68]. Soil application of sewage sludge increased the soil content of organic matter, which can explain its beneficial influence on the content of trace elements in the analysed aboveground biomass of oat.

The application of sewage sludge to soil contributed to the reduced phytoavailability of chromium, nickel, copper, manganese and cobalt in all treatments. As for lead and zinc, sewage sludge produced a contrary effect, leading to a significant increase in the content of these elements in aboveground biomass of oat. However, it only had a slight effect on cadmium. Moreover, the addition of sewage sludge to soil did not cause unidirectional changes in the content of iron in oat. The significant increase in the content of Pb and Zn in the aboveground biomass of oat was probably connected with its high content (especially zinc—849 mg kg^−1^ d.m.) in sewage sludge. Similar results were achieved by Wieczorek et al. [69], who—after incorporating sewage sludge to soil—observed a statistically significant decrease in nickel and copper in both the roots and the aboveground organs of maize. They also demonstrated that the enrichment of soil with zinc contained in sewage sludge I and II (77.4 mg and 49.9 mg pot^−1^, respectively) led to the higher accumulation of this element in maize roots accompanied by a statistically significant decrease in its content in shoots.

Bowszys et al. [70] showed that fertilisation with sewage sludge effected an increase in the content of available forms of copper and zinc in soil while not changing the soil nutrient abundance class (moderate for Zn and low for Cu). Pogrzeba et al. [71] proved the high effectiveness of a mixture of sewage sludge and fly ash from coal combustion in the immobilisation of lead, zinc and cadmium in soil under polluted arable land. The application of a mixture of these two types of waste also reduced the uptake of the three mentioned elements by *Festuca rubra* grass.

Sewage sludge is applied as an organic fertiliser, rich in ingredients which improve the biological, chemical and physical properties of soil. Sewage sludge can also be used in assisted phytostabilisation of trace elements, which eventually enables the formation of plant cover on polluted soil [72]. Grobelak et al. [73] concluded that a single application of sewage sludge was beneficial for the development of a plant cover, and after five years noticed a considerable increase in the biomass of trees, mainly spruce and pine. They also demonstrated that the content of cadmium, zinc and lead in the plant biomass was much lower than in the biomass of plants from the control plots.

Soil pollution with petroleum products creates a high threat to society and the environment [74] and also reduces the agricultural productivity of soils [75]. Therefore, research contributing to the understanding of changes occurring in real time in such an ecosystem is very valuable. The results of our research have contributed to a better understanding of the changes taking place in the chemical composition of plants cultivated on the soil polluted with diesel oil. Based on the results obtained, it can be concluded that the quality of soil contaminated with diesel oil and other petroleum products can be improved by using stabilised sewage sludge. The obtained results clearly indicate the need for a continuous search for and implementation of a bioremediation strategy of soil polluted with petroleum compounds. It will allow us to restore soils contaminated with petroleum products, which can then be used in agricultural production and will ensure food safety.

## 5. Conclusions

The impact of soil contamination with diesel oil on the content of trace elements in oat depended on the applied dose of the xenobiotic and the level of application of sewage sludge to soil. Increasing doses of diesel oil in the series without sewage sludge effected a decrease in the content of chromium, zinc, copper and cobalt as well as a higher accumulation of cadmium, lead, nickel, iron and manganese in aboveground organs of oat plants. In general, sewage sludge applied to soil for its remediation contributed to a decrease in quantities of elements accumulated in aboveground biomass of oat, except lead and zinc, whose content increased. Regardless its dose, the application of sewage sludge to soil had a slight effect on the average content of cadmium in the aboveground biomass of the test plant. Moreover, it did not cause consistent changes in the content of iron.

## Figures and Tables

**Table 1:** Physicochemical parameters of soil and sewage sludge applied in experiment.

Parameters	Unit	Soil	Sewage Sludge
Sand >0.05 mm	%	78.08	-
Silt 0.002–0.05 mm	%	20.46	-
Clay <0.002 mm	%	1.46	-
pH in 1 M KCl		6.58	8.20
Hydrolytic acidity	mM_(+)_ kg^−1^	8.50	-
Cation exchange capacity (CEC)	mM_(+)_ kg^−1^	105.0	-
Total exchangeable bases (TEB)	mM_(+)_ kg^−1^	113.5	-
Base saturation (BS)	%	92.5	-
Organic carbon (TOC)	g kg^−1^ d.m.	12.7	-
Total nitrogen	g kg^−1^ d.m.	0.88	51.0
Total phosphorus	g kg^−1^ d.m.	-	38.8
Available phosphorus	mg kg^−1^ d.m.	17.6	-
Available potassium	mg kg^−1^ d.m.	20.7	-
Total magnesium	g kg^−1^ d.m.	-	8.9
Available magnesium	mg kg^−1^ d.m.	8.8	-

**Table 2:** Content of cadmium (Cd), lead (Pb) and chromium (Cr) in aboveground parts of oat —*Avena sativa* L. (mg kg^−1^ d.m.).

Diesel Oil Dose (cm^3^ kg^−1^ d.m. of Soil)	Sewage Sludge Dose (g kg^−1^ d.m. of Soil)				Average
	0	4	8	12	
Cadmium (Cd)					
0	0.165 *^defg^*	0.131 *^g^*	0.151 *^fg^*	0.157 *^fg^*	0.151 *^A^*
5	0.186 *^bcdef^*	0.188 *^bcdef^*	0.161 *^efg^*	0.157 *^fg^*	0.173 *^B^*
10	0.202 *^abcd^*	0.209 *^abc^*	0.197 *^abcde^*	0.173 *^cdef^*	0.195 *^C^*
15	0.203 *^abcd^*	0.232 *^a^*	0.214 *^ab^*	0.235 *^a^*	0.221 *^D^*
Average	0.189 *^I^*	0.190 *^I^*	0.181 *^I^*	0.181 *^I^*	0.185
*r*	0.943 **	0.967 **	0.978 **	0.870 **	0.999 **
LSD for:	diesel oil dose (DO)—0.011, sewage sludge dose (SS)—0.011, interaction - 0.022				
Lead (Pb)					
0	0.246 *^de^*	0.188 *^e^*	0.197 *^e^*	0.227 *^e^*	0.215 *^A^*
5	0.251 *^cde^*	0.339 *^bcd^*	0.422 *^ab^*	0.348 *^bc^*	0.340 *^B^*
10	0.366 *^ab^*	0.401 *^ab^*	0.454 *^a^*	0.454 *^a^*	0.419 *^C^*
15	0.429 *^ab^*	0.401 *^ab^*	0.422 *^ab^*	0.435 *^ab^*	0.422 *^C^*
Average	0.323 *^I^*	0.332 *^I, II^*	0.374 *^III^*	0.366 *^II, III^*	0.349
*r*	0.954 **	0.900 **	0.768 **	0.910 **	0.931 **
LSD for:	diesel oil dose (DO)—0.027, sewage sludge dose (SS)—0.027, interaction—0.054				
Chromium (Cr)					
0	1.303 *^de^*	1.623 *^bc^*	2.177 *^a^*	1.303 *^de^*	1.602 *^A^*
5	1.535 *^bcd^*	1.307 *^cde^*	1.475 *^bcd^*	1.704 *^b^*	1.505 *^A^*
10	1.319 *^cde^*	1.046 *^ef^*	1.094 *^ef^*	0.485 *^g^*	0.986 *^B^*
15	0.926 *^f^*	0.241 *^g^*	0.204 *^g^*	0.293 *^g^*	0.416 *^C^*
Average	1.271 *^I^*	1.054 *^II^*	1.238 *^I^*	0.946 *^II^*	1.127
*r*	−0.687 **	−0.962 **	−0.989 **	−0.820 **	−0.964 **
LSD for:	diesel oil dose (DO)—0.087, sewage sludge dose (SS)—0.087, interaction—0.174				

Significant at: ** *p* ≤ 0.01, * *p* ≤ 0.05; r—correlation coefficient; values denoted by the different letters and Roman numerals are significantly different at *p* ≤ 0.01: *A*–*D* for diesel oil dose, *I*–*III* for sewage sludge dose and *a*–*g* for interaction between diesel oil dose and sewage sludge dose (Anova, Tukey’s HSD test).

**Table 3:** Content of nickel (Ni), zinc (Zn) and copper (Cu) in aboveground parts of oat—*Avena sativa* L. (mg kg^−1^ d.m.).

Diesel Oil Dose (cm^3^ kg^−1^ d.m. of Soil)	Sewage Sludge Dose (g kg^−1^ d.m. of Soil)				Average
	0	4	8	12	
Nickel (Ni)					
0	2.054 *^abc^*	1.668 *^bc^*	2.805 *^a^*	2.542 *^ab^*	2.267 *^A^*
5	2.094 *^abc^*	1.363 *^cd^*	1.941 *^abc^*	2.384 *^ab^*	1.946 *^BC^*
10	2.164 *^abc^*	2.477 *^ab^*	1.823 *^bc^*	1.823 *^bc^*	2.072 *^AB^*
15	2.269 *^ab^*	0.733 *^d^*	1.946a *^bc^*	1.786 *^bc^*	1.684 *^C^*
Average	2.145 *^I^*	1.560 *^II^*	2.129 *^I^*	2.134 *^I^*	1.992
*r*	0.980 **	−0.301	−0.766 **	−0.946 **	−0.858 **
LSD for:	diesel oil dose (DO)—0.242, sewage sludge dose (SS)—0.242, interaction—0.484				
Zinc (Zn)					
0	17.69 *^ab^*	18.92 *^a^*	16.43 *^abc^*	19.15 *^a^*	18.05 *^A^*
5	12.76 *^def^*	14.81 *^bcde^*	17.69 *^ab^*	16.07 *^abcd^*	15.33 *^B^*
10	10.23 *^f^*	12.24 *^f^*	13.62 *^cde^*	15.36 *^bcde^*	12.86 *^C^*
15	12.36 *^ef^*	12.28 *^ef^*	13.31 *^cdef^*	9.99 *^f^*	11.99 *^C^*
Average	13.26 *^I^*	14.56 *^II^*	15.26 *^II^*	15.14 *^II^*	14.56
*r*	−0.758 **	−0.924 **	−0.809 **	−0.956 **	−0.979 **
LSD for:	diesel oil dose (DO)—0.924, sewage sludge dose (SS)—0.924, interaction—1.848				
Copper (Cu)					
0	4.285 *^a^*	2.530 *^bc^*	2.949 *^b^*	4.426 *^a^*	3.548 *^A^*
5	2.621 *^bc^*	2.581 *^bc^*	2.377 *^bcd^*	1.268 *^ef^*	2.212 *^B^*
10	2.066 *^bcdef^*	2.258 *^bcde^*	1.958 *^bcdef^*	1.936 *^cdef^*	2.055 *^B^*
15	1.104 *^f^*	1.472 *^def^*	1.070 *^f^*	1.268 *^ef^*	1.229 *^C^*
Average	2.519 *^I^*	2.210 *^I, II^*	2.089 *^II^*	2.225 *^I, II^*	2.261
*r*	−0.977 **	−0.881 **	−0.988 **	−0.757 **	−0.956 **
LSD for:	diesel oil dose (DO)—0.276, sewage sludge dose (SS)—0.276, interaction—0.552				

Significant at: ** *p* ≤ 0.01; r—correlation coefficient; Values denoted by the different letters and Roman numerals are significantly different at *p* ≤ 0.01: *A*–*D* for diesel oil dose, *I*–*III* for sewage sludge dose and *a*–*g* for interaction between diesel oil dose and sewage sludge dose (Anova Tukey’s HSD test).

**Table 4:** Content of manganese (Mn), iron (Fe) and cobalt (Co) in aboveground parts of oat—*Avena sativa* L. (mg kg^−1^ d.m.).

Diesel Oil Dose (cm^3^ kg^−1^ d.m. of Soil)	Sewage Sludge Dose (g kg^−1^ d.m. of Soil)				Average
	0	4	8	12	
Manganese (Mn)					
0	44.05 *^i^*	39.29 *^j^*	42.35 *^ij^*	44.43 *^i^*	42.53 *^A^*
5	73.22 *^g^*	69.67 *^g^*	65.00 *^h^*	87.39 *^e^*	73.82 *^B^*
10	102.86 *^d^*	98.84 *^d^*	79.46 *^f^*	91.05 *^e^*	93.05 *^C^*
15	128.25 *^a^*	99.06 *^d^*	121.47 *^b^*	114.42 *^c^*	115.80 *^D^*
Average	87.10 *^I^*	76.72 *^II^*	77.07 *^II^*	84.32 *^III^*	81.30
*r*	0.999 **	0.944 **	0.976 **	0.946 **	0.995 **
LSD for:	diesel oil dose (DO)—1.186, sewage sludge dose (SS)—1.186, interaction—2.372				
Iron (Fe)					
0	70.40 *^efg^*	120.87 *^a^*	92.54 *^b^*	78.37 *^de^*	90.55 *^A^*
5	75.36 *^def^*	66.01 *^fg^*	90.18 *^bc^*	81.12 *^cde^*	78.17 *^B^*
10	91.88 *^bc^*	90.98 *^bc^*	75.80 *^def^*	75.04 *^defg^*	83.43 *^C^*
15	83.01 *^bcd^*	53.48 *^h^*	64.38 *^g^*	92.39 *^b^*	73.32 *^D^*
Average	80.16 *^I^*	82.84 *^I^*	80.73 *^I^*	81.73 *^I^*	81.36
*r*	0.748 **	−0.769 **	−0.969 **	0.617 *	−0.812 **
LSD for:	diesel oil dose (DO)—2.972, sewage sludge dose (SS)—2.972, interaction—5.944				
Cobalt (Co)					
0	3.398 *^a^*	3.212 *^abc^*	2.636 *^de^*	2.552 *^de^*	2.950 *^A^*
5	3.233 *^ab^*	3.224 *^abc^*	3.215 *^abc^*	2.928 *^bcd^*	3.150 *^B^*
10	2.823 *^cde^*	2.636 *^de^*	2.742 *^de^*	2.916 *^bcd^*	2.779 *^C^*
15	2.791 *^de^*	2.922 *^bcd^*	2.698 *^de^*	2.418 *^e^*	2.707 *^C^*
Average	3.061 *^I^*	2.999 *^I^*	2.823 *^II^*	2.704 *^II^*	2.897
*r*	−0.955 **	−0.674 **	−0.140	−0.207	−0.719 **
LSD for:	diesel oil dose (DO)—0.112, sewage sludge dose (SS)—0.112, interaction—0.224				

Significant at: ** *p* ≤ 0.01, * *p* ≤ 0.05; r—correlation coefficient; values denoted by the different letters and Roman numerals are significantly different at *p* ≤ 0.01: *A*–*D* for diesel oil dose, *I*–*III* for sewage sludge dose and *a*–*g* for interaction between diesel oil dose and sewage sludge dose (ANOVA, Tukey’s HSD test).

**Table 5:** Correlation coefficients (*r*) between content of trace elements in aboveground parts of oat—*Avena sativa* L.

Trace Elements	Pb	Cr	Ni	Zn	Cu	Mn	Fe	Co
Cd	0.604 **	−0.724 **	−0.419 **	−0.786 **	−0.475 **	0.766 **	−0.400 **	−0.384 **
Pb		−0.672 **	−0.245	−0.506 **	−0.634 **	0.770 **	−0.311 *	−0.239
Cr			0.489 **	0.468 **	0.467 **	−0.701 **	0.443 **	0.287 *
Ni				0.116	0.257	−0.155	0.334 *	−0.235
Zn					0.609 **	−0.779 **	0.204	0.385 **
Cu						−0.794 **	0.081	0.305 *
Mn							−0.268	−0.395 **
Fe								−0.031

Significant at ** *p* ≤ 0.01, * *p* ≤ 0.05.

## Data Availability

Data are available by contacting the authors.
